# The role of heterogenous environmental conditions in shaping the spatiotemporal distribution of competing *Aedes* mosquitoes in Panama: implications for the landscape of arboviral disease transmission

**DOI:** 10.1007/s10530-021-02482-y

**Published:** 2021-03-01

**Authors:** Kelly L. Bennett, W. Owen McMillan, Vanessa Enríquez, Elia Barraza, Marcela Díaz, Brenda Baca, Ari Whiteman, Jaime Cerro Medina, Madeleine Ducasa, Carmelo Gómez Martínez, Alejandro Almanza, Jose R. Rovira, Jose R. Loaiza

**Affiliations:** 1 Smithsonian Tropical Research Institute, Balboa Ancón, Republic of Panama; 2 The University of Texas, El Paso, TX USA; 3 Universidad del Atlántico, Barranquilla, Colombia; 4 Programa Centroamericano de Maestría en Entomología, Universidad de Panamá, Panamá, República de Panamá; 5 Instituto de Investigaciones Científicas Y Servicios de Alta Tecnología, Panamá, República de Panamá

**Keywords:** *Aedes* mosquitoes, Arbovirus transmission, Interspecific competition, Ecological niche, Environmental gradient, Geographic species displacement, Panama

## Abstract

Monitoring the invasion process of the Asian tiger mosquito *Aedes albopictus* and its interaction with the contender *Aedes aegypti,* is critical to prevent and control the arthropod-borne viruses (i.e., Arboviruses) they transmit to humans. Generally, the superior ecological competitor *Ae. albopictus* displaces *Ae. aegypti* from most geographic areas, with the combining factors of biology and environment influencing the competitive outcome. Nonetheless, detailed studies asserting displacement come largely from sub-tropical areas, with relatively less effort being made in tropical environments, including no comprehensive research about *Aedes* biological interactions in Mesoamerica. Here, we examine contemporary and historical mosquito surveillance data to assess the role of shifting abiotic conditions in shaping the spatiotemporal distribution of competing *Aedes* species in the Republic of Panama. In accordance with prior studies, we show that *Ae. albopictus* has displaced *Ae. aegypti* under suboptimal wet tropical climate conditions and more vegetated environments within the southwestern Azuero Peninsula. Conversely, in the eastern Azuero Peninsula, *Ae. aegypti* persists with *Ae. albopictus* under optimal niche conditions in a dry and more seasonal tropical climate. While species displacement was stable over the course of two years, the presence of both species generally appears to fluctuate in tandem in areas of coexistence. *Aedes albopictus* was always more frequently found and abundant regardless of location and climatic season. The heterogenous environmental conditions of Panama shape the competitive outcome and micro-geographic distribution of *Aedes* mosquitoes, with potential consequences for the transmission dynamics of urban and sylvatic zoonotic diseases.

**Supplementary Information:**

The online version of this article (10.1007/s10530-021-02482-y).

## Introduction

In the modern era, the invasion of insect vectors is often associated with long distance commodity trade, including various examples of human assisted-dispersal by ships, airplanes and terrestrial vehicles (Lounibos and Kramer 2016). One important example is the global expansion and ecological success of *Aedes* (*Stegomyia*) *albopictus* (Skuse) and *Aedes* (Stegomyia) *aegypti* (L.) mosquitoes, for which pre-adaptation to human commensalism has promoted their ability to disperse via our transport networks. Previous research has recognized that the quality and quantity of larval habitats driving *Aedes* abundance, plus the knowledge and actions to prevent breeding and biting, vary as function of the ecologic, demographic and socioeconomic conditions in which human populations live (LaDeau et al. 2013; Whiteman et al. 2019b, a). The eggs of *Aedes* (Stegomyia) spp. have evolved hydrophobic outer layers to remain viable for months even while the surrounding water habitat has dried, allowing them to hatch when rain returns. This strategy allows mosquitoes in natural tree-hole habitats to persist through the dry season, whilst in anthropogenic settings they can resist desiccation inside artificial containers such as used-tires (Rose et al. 2020). Accordingly, not only is the development and survival of *Ae. albopictus* and *Ae. aegypti* influenced by human behaviour, but people also offer transportation routes and commodities for these vectors to invade novel geographic areas.

Invasive *Ae. albopictus* and *Ae. aegypti* are ecological rivals whose global distribution has shifted over the past decades owing to differences in their abilities to biologically outcompete one another. Widespread displacement of *Ae. aegypti* by *Ae. albopictus* has occurred, with the combining factors of biology and environment influencing the competitive outcome (O’meara et al. 1995; Braks et al. 2003; Kaplan et al. 2010; Bagny Beilhe et al. 2012; Hopperstad and Reiskind 2016; Muzari et al. 2019). A potential mechanism of interspecific competition in *Aedes* includes larval competition for space and food resources, with *Ae. albopictus* generally being the superior competitor under experimental conditions and found to impact on the longevity, development time and survival of *Ae. aegypti* (Juliano 1998; Braks et al. 2004; Reiskind and Lounibos 2009; Murrell and Juliano 2014; Lounibos and Juliano 2018). Another mechanism is mating interference, where satyrization after interspecific mating leads to infertile *Ae. aegypti* females through the action of accessory gland products, but which does not impact on *Ae. albopictus* (Bargielowski et al. 2016). Studies from Florida in the USA, Southeastern Brazil, Bermuda, Mayotte and the Torres Strait of Australia have documented a decline in the range and abundance of *Ae. aegypti* after invasion by *Ae. albopictus* (Braks et al. 2003; Kaplan et al. 2010; Bagny Beilhe et al. 2012; Hopperstad and Reiskind 2016; Muzari et al. 2019). Nonetheless, while studies across the world have reported a similar outcome of species displacement, in Florida and Southeastern Brazil, for example, *Ae. aegypti* persists in both urban and warm/dry environments despite species displacement by *Ae. albopictus* throughout much of its previous geographical range (Braks et al. 2003).

The outcome of competitive interaction between *Aedes* (Stegomyia) spp. appears to be condition dependant on both the environment and level of urbanicity (Braks et al. 2003; Reiskind and Lounibos 2013; Hopperstad and Reiskind 2016; Hopperstad et al. 2020). Indeed, mediation of competition by climate was suggested earlier as a mechanism for rapid displacement in these mosquitoes, but this has been proposed mainly for areas where tropical *Ae. aegypti* is at its ecological limit in terms of tolerance to winter temperatures (e.g., in subtropical Florida and Bermuda), which could give the advantage to a temperate species like *Ae. albopictus*. Furthermore, while a recent study in tropical Mayotte showed a significant decrease in the proportion of sites occupied by *Ae. aegypti* and an increase of *Ae. albopictus* in urban and suburban zones, it failed to assess the impact of climatic variability as a predictor of local species distributions (Bagny Beilhe et al. 2012). Therefore, more empirical evidence is needed to better understand the early stages of the invasion dynamic and the robustness of these findings in tropical areas. *Aedes albopictus* and *Ae. aegypti* have different competencies to transmit arboviruses. While *Ae. albopictus* is a biologically competent vector for a broader spectrum of viral pathogens, including sylvatic zoonotic agents (e.g., animal origin), the majority of dengue virus serotypes in urban centers are only efficiently transmitted by *Ae. aegypti* (Pereira Dos Santos et al. 2018). Therefore, ongoing biotic interactions between *Ae. aegypti* and *Ae. albopictus* could have important consequences for arbovirus transmission, including a decrease in urban disease prevalence and an increase in sylvatic disease emergence to humans or both. Evidence combined would indicate that the risk of arbovirus epidemics can only be estimated by decoding the link between *Aedes* species occurrence in a heterogenous environmental, demographic and socioeconomic landscape.

Despite increasing efforts to better understand the ecological interaction between *Ae. albopictus* and *Ae. aegypti* around the world, we still know little as to how micro-geographic environmental variability influences their distribution across space and time. Specifically, we do not know how contrasting environmental conditions across natural-anthropogenic landscapes can shape their local patterns of displacement or coexistence. Although there are some detailed studies asserting displacement within sub-tropical areas (Kaplan et al. 2010; Bargielowski et al. 2016; Lounibos and Juliano 2018), there has been no comprehensive research about the biological interaction of *Aedes* species done in Mesoamerica. Analogous to other geographical regions of the world (Bonizzoni et al. 2013), *Ae. albopictus* has been precipitously spreading throughout Panama since its introduction in 2002. The Asian tiger mosquito expanded rapidly across the country likely assisted by the transportation of used-tires, reaching both urban regions and isolated rural areas, where it began interacting with resident *Ae. aegypti* with potential consequences for the local disease landscape (Miller and Loaiza 2015; Eskildsen et al. 2018; Bennett et al. 2019a; Whiteman et al. 2019a, b). At present no efforts have been made by local health authorities to understand changes in the geographic distribution of resident *Ae. aegypti* after the invasion of *Ae. albopictus*. Attaining a better understanding of the dynamics of species biological displacement across Panama may improve the capacity of public health authorities to combat the spread of sylvatic yellow fever (YF), Mayaro (MAY), and Venezuelan Equine Encephalitis (VEEV), urban dengue (DENV), and emerging West Nile (WN), chikungunya (CHIKV) and Zika (ZIKV) arboviruses.

Encompassing a period of increasing inter-specific competition between *Ae. aegypti* and *Ae. albopictus* in lower Mesoamerica, Panama’s vector surveillance system is particularly unique and useful to study the biotic interaction between *Aedes* vectors (Whiteman et al. 2019b). Herein, we test the effect that heterogenous environmental conditions across Panama have on the spatiotemporal distribution of competing *Aedes* mosquitoes. Specifically, we examine the stability of species associations in the Azuero Peninsula, an isolated region of central Panama subject to a sharp West to East environmental gradient (i.e., > 150 km) moving from wet tropical conditions to dry tropical conditions. *Aedes albopictus* invaded the Azuero Peninsula for the first time in 2014, where resident *Ae. aegypti* had been recorded as the sole vector species since 1973. Therefore, the Azuero Peninsula provides ideal conditions to test whether or not *Ae. aegypti* has been displaced by *Ae. albopictus* from across a shifting environment in a short period of time. We employ a non-experimental field design along with systematic mosquito sampling from across the entire country of Panama to identify the environmental variables that impact on *Aedes* species presence and absence through the use of both non-spatial and spatial correlative analytical approaches of species distribution.

## Materials and Methods

### Mosquito data and environmental variables

**Fig. 1:**
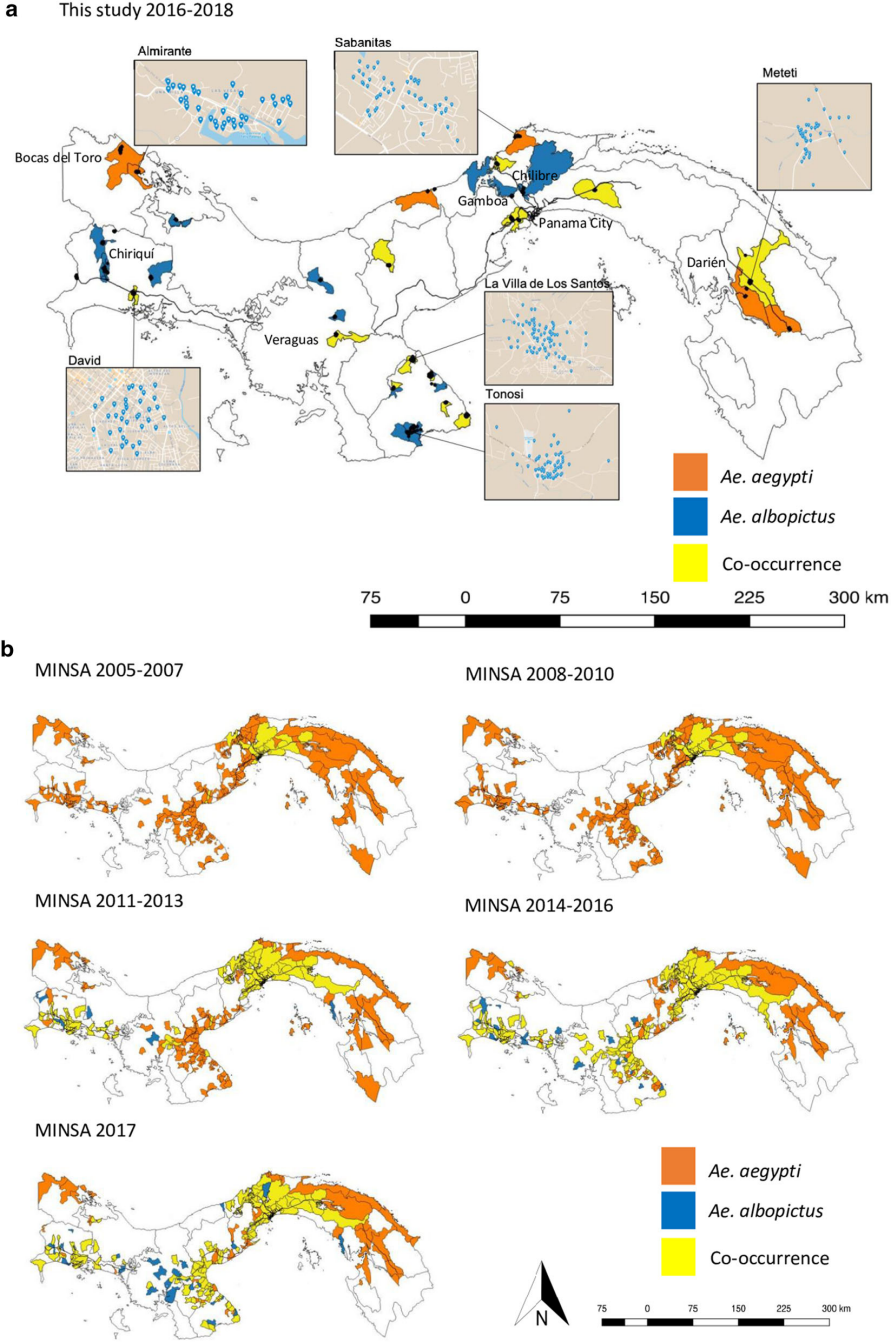
**a** The presence of *Ae*. *aegypti* (orange corregimientos, i.e. counties), *Ae. albopictus* (blue corregimientos) and species co-occurrence (yellow corregimientos) recorded by extensive sampling with oviposition traps during the wet season months from 2016 through to 2018 in comparison to **b** Species occurrence data recorded from 2005 through 2017 through active surveillance by the Ministry of Health in Panama. Provinces are indicated by the black boundaries

**Fig. 2:**
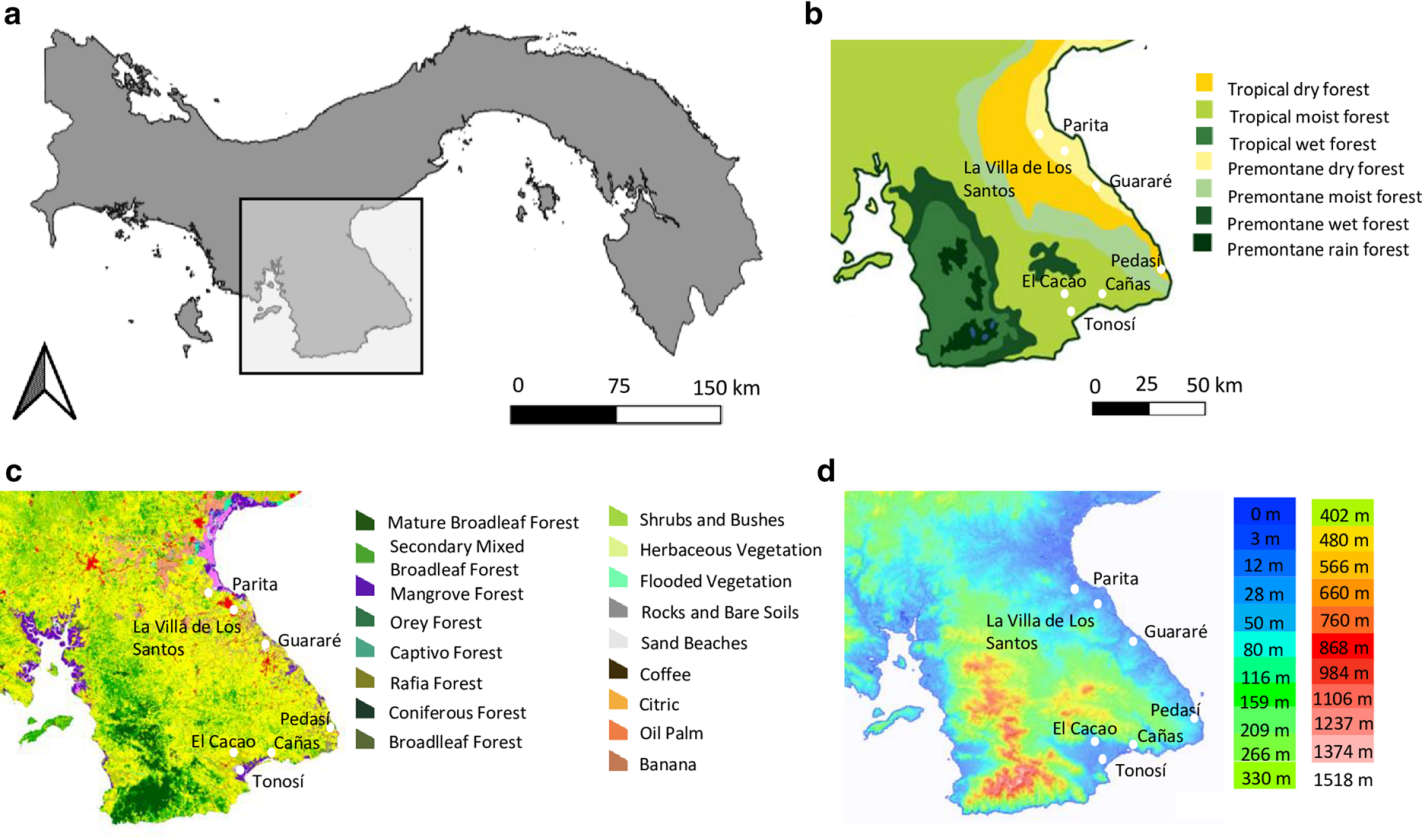
Sampling locations in the **a** Azuero Peninsula of Panama in relation to **b** life zones based on Holdridge (1967) (map modified is from the Smithsonian Tropical Research Institute www.stri.si.edu) **c** the underlying forest cover and land use of the area (sourced from the open source STRI GIS data portal https://stridata-si.opendata.arcgis.com) and **d** topography and altitude (map modified from the open source site topographic-map.com)

To investigate both the geographical distributions and ecological niche of *Ae. aegypti* and *Ae. albopictus*, mosquitoes were collected using oviposition traps placed across 951 observation points, 35 settlements and nine provinces of Panama during the rainy season months of May to November from 2016 to 2018 (Fig. 1a and Supplementary Table S1 online). In addition, from 2018 to 2019 we gathered high resolution (i.e., through frequent and periodic sampling within the same locations) regional and temporal data on species presence and absence acquired through the sampling of three locations in the southwest and four locations in the East Azuero Peninsula (Fig. 2 and Supplementary Table S1). All these localities fall within a similar range of values for land use type, topography, altitude, human demography and were connected through a major northeast southwest highway to avoid bias due to human-aided dispersal or differences in the level of urbanicity. This data was collected monthly throughout both the rainy and dry seasons of the two consecutive years.

We also utilized entomological surveillance data from the Vector Control Department (VCD) at the Panamanian Ministry of Health (Ministerio de Salud de la República de Panamá—MINSA) (http://www.minsa.gob.pa) collected from 2005 to 2017. Systematic mosquito collections have occurred in Panama in order to establish *Aedes* infestation rates, and hence, risky areas for dengue transmission. Surveys of both *Ae. aegypti* and *Ae. albopictus* are performed annually at the Corregimiento-scale (i.e., county), involving mainly larval surveillance. Each year, a random block of houses is chosen and all houses in the block are searched for containers holding *Aedes* larvae. The larvae are collected and allowed to mature to the fourth instar, at which point they are taxonomically identified to species based on morphological keys (Rueda 2004). The number of houses positive for *Ae. aegypti*, *Ae. albopictus* or both are recorded in the raw datasets. Since we cannot confirm the number of houses in each block, we have transformed the data into a presence-absence format in each Corregimiento rather than analyzing the number of positive houses or the number of mosquitoes collected per house as in Whiteman et al. (2019b).

Climate variables representing 10 years of averaged data from ~ 60 meteorological stations across Panama included average rainfall, average humidity, average minimum temperature and average maximum temperature. In addition, normalized difference vegetation indexes (NDVI) obtained from the National Aeronautics and Space Administration (NASA, USA) and human population density values obtained from the Instituto Nacional de Estadística y Censo 2010 (https://www.inec.gob.pa) were included in the analysis. The environmental data for each sampling point were extracted from each environmental raster layer in QGIS (QGIS Development Team 2019). Additional details on the collection, selection and processing of the environmental data for Panama are given in Bennett et al. (Bennett et al. 2020c).

Furthermore, to determine whether environmental differences are observed between mosquito species on a micro-habitat scale, *Aedes* mosquitoes were collected from natural and artificial oviposition sites, mainly comprising tires and water containers, and were reared in the laboratory for species identification. At each of the 158 oviposition sites, the water temperature and water pH were measured three times, and an average measurement recorded (Supplementary Table S2).

### Data analysis

**Fig. 3:**
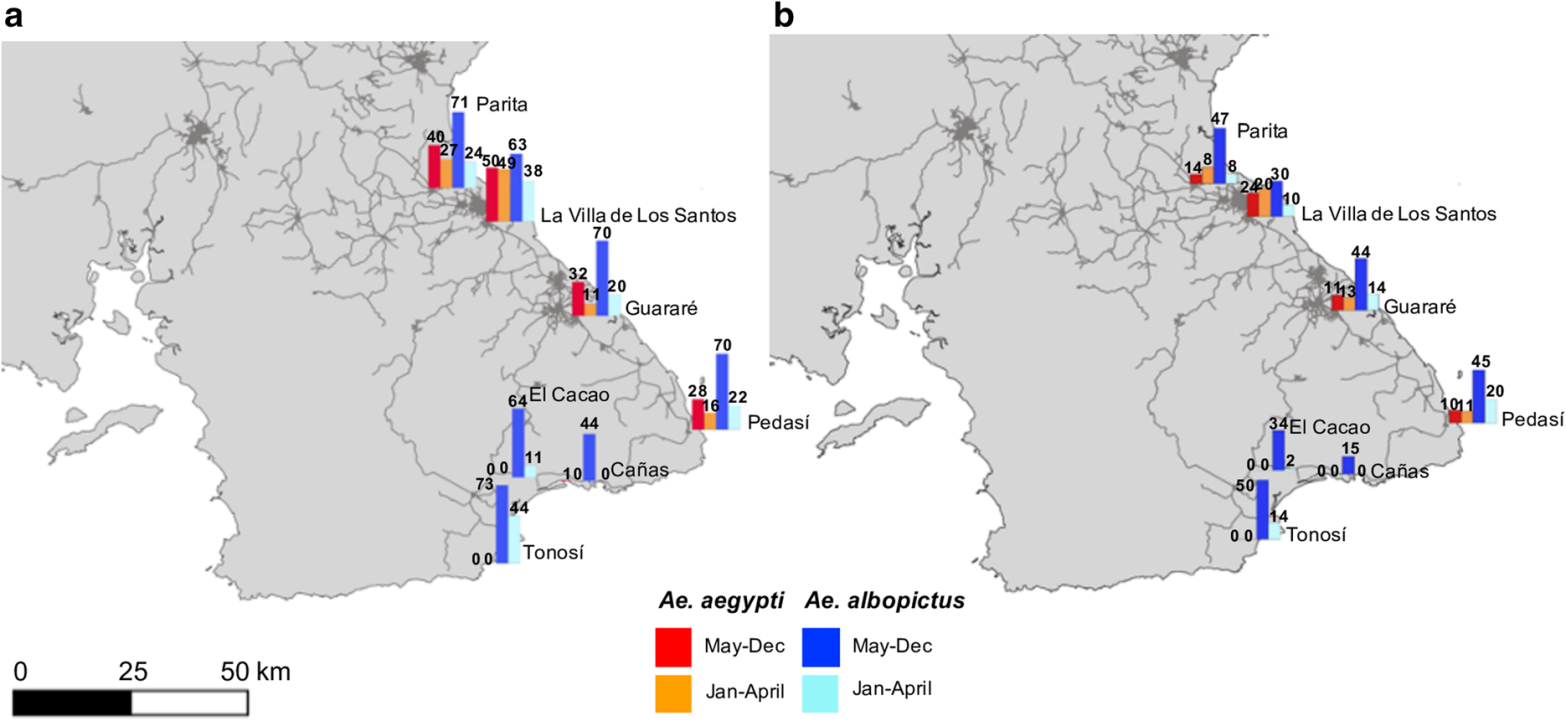
**a** The proportion of sampled sites positive for *Ae. aegypti* and *Ae. albopictus* and **b** the median abundance of each species at each location across the dry (January–April) and wet (May–December) season months. The network of highways and roads across the Azuero Peninsula is shown in solid dark lines

First, we produced maps representing the species distribution of *Ae. aegypti* and *Ae. albopictus* across Panama from 2005 to 2018 in QGIS (QGIS Development Team 2019) using our mosquito surveillance data widespread across the country (i.e., 951 observation points, 35 settlements and nine provinces during the rainy seasons of 2016, 2017, and 2018) and that obtained from MINSA (i.e., 14 years of historical data from 462 localities, 63 districts and nine provinces). The proportion of sampling sites positive for *Ae. aegypti* and *Ae. albopictus* presence from 2005 through 2018 were calculated. Second, we analyzed the fine-scale regional data comprising two years of mosquito collections from the Azuero Peninsula. We calculated the proportion of oviposition traps that were positive for either species at each sampling locality and plotted the data as a bar chart for the East and Southwest Azuero Peninsula for each month (Supplementary Fig. S1 online) and for the dry and rainy seasons (Fig. 3a). The median abundance of each species across the Azuero Peninsula was also plotted for the same time frame (Fig. 3b). Third, we used all the mosquito surveillance data sets (i.e., historical *Aedes* collections from MINSA, our systematic sampling across Panama, plus focal data from the Azuero Peninsula) to acquire species distribution predictions for *Ae. aegypti* and *Ae. albopictus* from across the entire country using Ensemble models of niche distributions. Finally, using all data, we conducted non-spatial statistical analysis to disentangle the factors influencing *Aedes* species displacement and persistence in different regions of the country.

### Niche distribution analyses

Ensemble models of species niche distributions are often used to minimize the impact of model performance variability across different species, regions and datasets, since there is no one method that consistently outperforms those available (Pearson et al. 2006; Hao et al. 2019). The approach we take combines the niche models with the best performance (i.e. greater accuracy) for the dataset, resulting in an averaged and weighted model according to each model’s predictive performance, which has been shown to improve ensemble predictions (Marmion et al. 2009; Thuiller et al. 2009). Raster layers were created for each environmental variable in QGIS as in Bennett et al. (QGIS Development Team 2019; Bennett et al. 2020c). A resolution of 0.05 pixels was used to better reflect the spatial resolution of the climatic data obtained from meteorological stations across Panama. Raster layers were imported and stacked in R (R Core Team 2018). For each mosquito species, and all the vector surveillance data, niche distribution models were projected using the Biomod2 ensemble method implemented in an R package (Thuiller et al. 2009). The selection of pseudo-absence data was not required given that more absence points were sampled than records of species presence. Six models with different underlying algorithms were run with the default parameter settings and with 20 replications each. These included Generalized Linear Models (GLM), Generalized Additive Models (GAM), Random Forests (RF), Maximum Entrophy (MAXENT, Phillips), Generalized Boosted Models (GBM) and Classification Tree Analysis (CTA) using 70% of the data, with the other 30% used to test model performance. Model performance was assessed with the true skill statistic (TSS) and the receiver operating characteristic curves (ROC), which take into account both the model sensitivity and specificity (Allouche et al. 2006; Thuiller et al. 2009). The highest performing models with TSS values over 0.5 were averaged and weighted based on the predictive accuracy of each model (Thuiller et al. 2009) and with equal weight given to the presence and absence data. Habitat probability maps for the resulting niche distribution models were created using Biomod2 (Thuiller et al. 2009).

We further used the R package Ecospat to perform comparative niche analyses (Di Cola et al. 2017). The niche overlap of *Ae. aegypti* and *Ae. albopictus* was calculated using Schoener's D index (Schoener 1968). A niche equivalency and niche similarity test (Warren et al. 2008) were performed using 1000 replications. These tests respectively use two distinct niche similarity statistics, *I* and *D*, to evaluate whether the species niche overlap is higher than expected by chance and whether the niche of *Ae. albopictus* predicts that of *Ae. aegypti* better than expected by chance taking into account the local environmental heterogeneity (Warren et al. 2008). We also quantified the niche dynamics of *Ae. aegypti* and *Ae. albopictus* with each environmental variable gradient using the built-in package function.

### Non-spatial analyses of species distributions

We first performed a Likelihood-Ratio Chi-Square test to determine whether the variables, mosquito presence and location, were independent or associated. Second, in order to determine which environmental variables impact on species geographical distributions, we used a generalized linear model statistical framework, i.e., GLM (McCullagh and Nelder 1972) implemented in the Stats R Package (R Core Team 2018) with a binomial linkage to test whether the presence of either *Ae. aegypti*, *Ae. albopictus* or the co-occurrence of both species was associated with the environmental predictors. For the former two tests, we also included the presence of an *Aedes* competitor as a predictor (i.e., the presence of either *Ae. aegypti* or *Ae. albopictus*). GLMs are robust and capable of being applied to data without homogeneous variance or normality. They have been utilized in a variety of studies on the public health implications of *Aedes* mosquito ecology (Carbajo et al. 2006; Chansang and Kittayapong 2007; Wang et al. 2011). Finally, we also evaluated the effect of water temperature and water pH from larval samples on species presence and absence using separate GLM runs for *Ae. aegypti* and *Ae. albopictus*.

## Results

### Spatial and temporal patterns of species distributions

To understand how the recent introduction of *Ae. albopictus* has shaped populations of *Ae. aegypti* across Panama over the last decade, we coupled historical surveys of mosquito populations with intensive sampling of focal populations. Over the sampling period, there has been significant changes in the geographic distribution of *Ae. aegypti* across Panama (Fig. 1). Although both species now co-exist in many regions throughout Panama, areas in the wet and humid Southwest Azuero Peninsula, rural Chiriquí, Veraguas and the province of Panamá outside of Panama City (Gamboa and Chilibre), were solely inhabited by *Ae. albopictus*. This includes regions, from which *Ae. aegypti* was previously documented by the health authorities, suggesting that *Ae. albopictus* has displaced *Ae. aegypti* in these areas. Although the distribution data from MINSA is generally in agreement with ours, discrepancies between the 2017 data and our data from 2016 to 2018, which show the co-existence of both *Aedes* within some corregimientos rather than *Ae. albopictus* alone, are likely due to the method of surveillance used. Active surveillance by MINSA is dependent on search effort, correct species identification and the financial resources available from year to year. The displacement of *Ae. aegypti* by *Ae. albopictus* was further supported by a general decrease in the proportion of positive sampling sites. This proportion has decreased for *Ae. aegypti* since 2005 from ~ 50% to ~ 20%, while the presence of *Ae. albopictus* has increased from 0 to ~ 65% (Supplementary Fig. S2 online). *Aedes aegypti* continued to be found in high abundance in Bocas del Toro and Darién, where *Ae. albopictus* has only recently arrived (Darién) or has not yet been documented (Bocas del Toro).

*Aedes aegypti* and *Ae. albopictus* are present across both the rainy and dry season months in the rural locations of Parita, La Villa de Los Santos, Guararé, and Pedasí in the eastern Azuero Peninsula (Fig. 3 and Supplementary Fig. S1 online). However, only *Ae. albopictus* is found within the rural localities of Cañas, El Cacao and Tonosí in the Southwest Azuero Peninsula throughout the entire year. In areas of coexistence, both species are present in a greater frequency during the rainy season months of May to December than during the dry season months of January to April. The presence of both species generally appears to fluctuate in tandem (Supplementary Fig. S1 online), but *Ae. albopictus* is always more frequently found and abundant regardless of location and climatic season (Fig. 3).

### Comparative niche prediction of *Aedes* mosquitoes

**Fig. 4:**
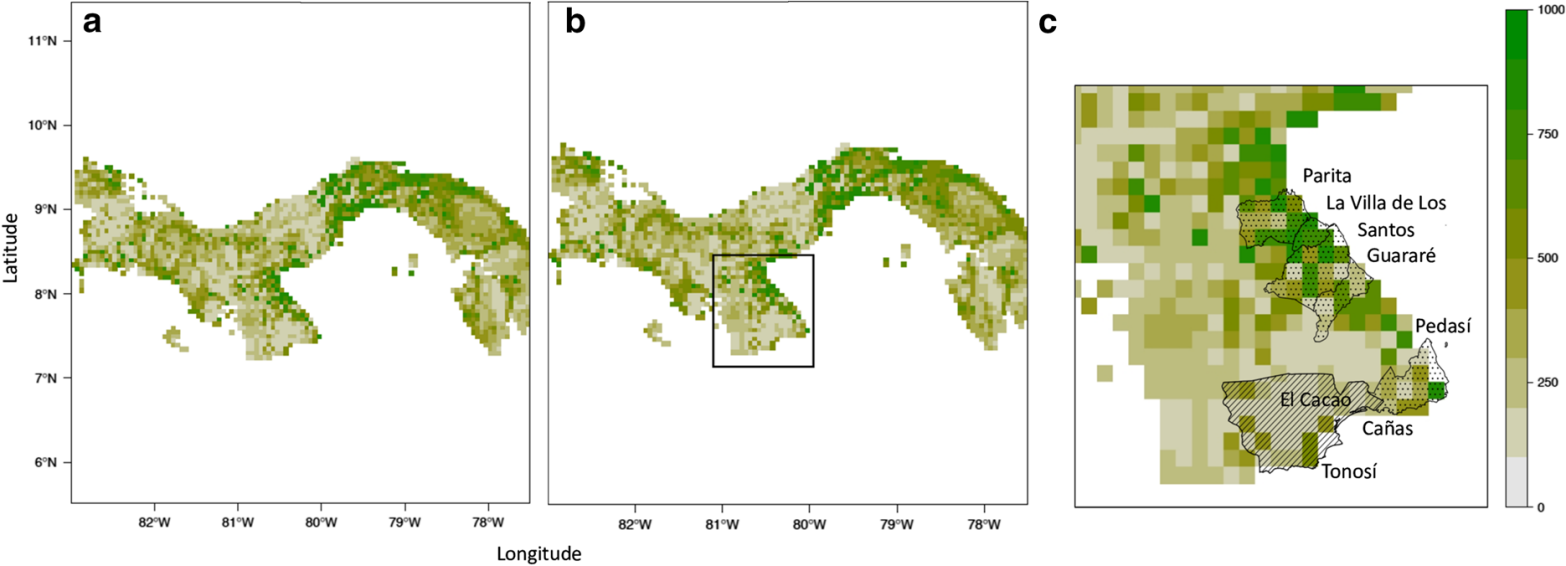
Weighted ensemble species niche distribution map for **a** *Ae. aegypti* and **b** *Ae. albopictus* showing habitat suitability across Panama with higher values indicating higher suitability. The region of the Azuero Peninsula is indicated in **b**. The niche distribution map for *Ae. albopictus* is shown in detail for the Azuero Peninsula in **c**. The map indicates areas where only *Ae. albopictus* was documented (hashed fill) and areas where both *Ae. aegypti* and *Ae. albopictus* were found (dotted fill) by the present study

The niche simulations with the best model performance were Random Forest (RF) and Classification Tree Analysis (CTA) with TSS values greater than 0.5 and ROC values greater than 0.80 for both species. The ensemble models for each species had TSS values of 0.58 while the ROC value for both species was 0.88. Ensemble niche distribution maps for both species on comparison were highly similar, with the most suitable environmental conditions seemingly occurring in the most populated regions of Panama, including the Caribbean city and province of Colón, Panama City and provincial Panamá, the central Pacific coastal region connecting provincial Panama to Coclé, the East Azuero Peninsula, the Pacific region surrounding the western city of David and the more densely inhabited regions of Bocas del Toro province on the western Caribbean coast (Fig. 4). For both *Ae. aegypti* and *Ae. albopictus*, all the environmental variables contributed to the chosen models, although human population density and rainfall were the most important in all models (Supplementary Table S3). Regions of species co-existence of *Ae. aegypti* and *Ae. albopictus* in the Azuero Peninsula occurred in areas classified by the niche distribution model as highly suitable whereas areas where *Ae. albopictus* was found alone were of lower suitability (Fig. 4).

**Fig. 5:**
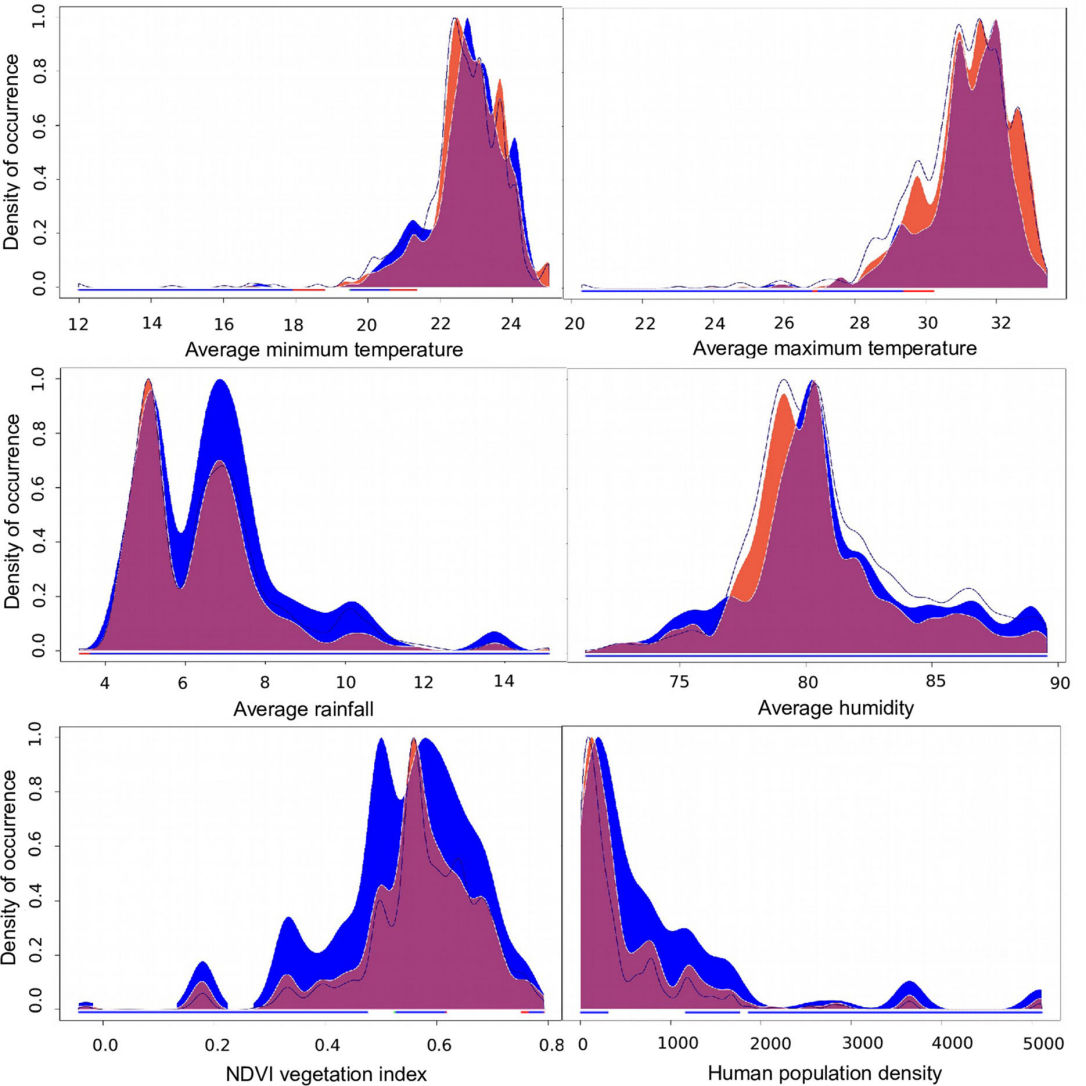
Density of occurrence of avaliable climates in the range of *Ae. aegypti* (red) and *Ae. albopictus* (blue) with niche overlap indicated by the purple area. The avaliable environment is shown as a dark blue line (*Ae. albopictus*) and dark red line (*Ae. aegypti*)

Niche overlap analysis revealed that *Ae. aegypti* and *Ae. albopictus* share 70% of their environmental niche. The niche equivalency test was non-significant indicating that the suitable climatic niche for these two species is not statistically different, using both the niche similarity statistics *I* (I = 0.905, *P* = 1.000) and *D* (D = 0.700, *P* = 1.000) (Warren et al. 2008). The niche similarity test further agreed with these findings, with significant values for *I* (I = 0.905, *P* = 0.001) and *D* (D = 0.700, *P* = 0.001), revealing that the two *Aedes* niches were more similar than expected by chance. A significant result here indicates that observed differences between the species are related to habitat availability rather than underlying habitat preferences. The niche dynamics analysis further revealed that *Ae. aegypti* and *Ae. albopictus* both occur across the same range of values for each environmental variable (Fig. 5). However, the two *Aedes* exhibit subtle differences in their niche concerning the climatic variables. For example, the probability of encountering *Ae. albopictus* increases with higher average rainfall and average humidity values while *Ae. aegypti* is found more at higher average maximum temperatures. *Aedes albopictus* populations are found at a higher density of occurrence than *Ae. aegypti* across the entire range of NDVI vegetation indexes and human population densities.

### Non-spatial analyses of species distributions

The Likelihood Chi-squared test revealed that the frequency of *Aedes* species occurrence and location are associated variables (*χ*_2_ = 262, df = 6, *P* < 0.01). Furthermore, the GLM showed that the presence of *Ae. aegypti* is negatively and significantly predicted by NDVI vegetation index (Z = -8.573, *P* < 0.01), average rainfall (Z =  − 5.551, *P* < 0.01) and average humidity (Z =  − 4.398, *P* < 0.01) while positively predicted by average minimum temperature (Z = 7.556, *P* < 0.01), and human population density (Z = 7.047, *P* < 0.01). Average maximum temperature was not a significant factor impacting the geographic distribution of *Ae. aegypti*. In addition, *Ae. aegypti* is positively associated with the presence of its competitor, *Ae. albopictus*, reflecting that both species coexist across much of their range (Z = 16.402, *P* < 0.01). Similarly, the GLM revealed that the presence of *Ae. albopictus* is negatively and significantly predicted by NDVI vegetation index (Z =  − 2.009, *P* < 0.05), while positively predicted by average rainfall (Z = 2.459, *P* < 0.05), average minimum temperature (Z = 5.799, *P* < 0.01), average humidity (Z = 6.334, *P* < 0.01), human population density (Z = 15.582, *P* < 0.01), and the presence of *Ae. aegypti *(Z = 16.727, *P* < 0.01). As in the case of *Ae. aegypti*, maximum temperature was not a significant variable predicting the presence of *Ae. albopictus*. The co-occurrence of both *Ae. aegypti* and *Ae. albopictus* is significantly associated with lower NDVI vegetation index (Z =  − 5.665, *P* < 0.01) increasing values of average minimum temperature (Z = 6.539, *P* < 0.01) and a higher human population density (Z = 16.104, *P* < 0.01). Average rainfall, average maximum temperature and average humidity do not impact species co-occurrence.

The GLM to determine whether the presence or absence of either species is impacted by water temperature or pH was borderline or not significant for *Ae. aegypti* (*Z* = 1.94, *P* = 0.05 and Z = -1.82, *P* = 0.07, respectively) while only water temperature was significant for *Ae. albopictus* (*Z* =  − 2.20, *P* = 0.03 and Z = 1.15, *P* = 0.25, respectively). However, the GLM was only borderline significant for rubber tires (*Z* =  − 1.97, *P* = 0.05) but not plastic containers (*Z* =  − 0.80, *P* = 0.42) when analyzed separately.

## Discussion

Our findings from the Azuero Peninsula and more broadly from across Panama confirm that *Ae. albopictus* is rapidly displacing *Ae. aegypti* throughout certain regions. Our data extend on previous findings to show that *Ae. albopictus* has displaced *Ae. aegypti* on the southwestern side of the Azuero Peninsula of Panama in less than 5 years, and that this extirpation is stable throughout time including both the rainy season and dry season months. Species displacement is further supported by historical evidence showing that *Ae. aegypti* once inhabited areas where only *Ae. albopictus* is found in the Southwest Azuero Peninsula and other wet tropical regions of Panama, where it is no longer present, including provincial Chiriquí, Veraguas and Panamá moving towards the Caribbean coast ~ 10 years ago.

Species displacement occurs because the two species share a similar ecological niche, suggesting that interspecific competition mainly drives the patterns of species distributions. Our niche modelling and statistical analysis of species distributions revealed no significant difference in either species fundamental niche, suggesting that both species should theoretically be able to persist together. We also found weak evidence of inter-species differences in micro-habitat usage. Although we found a significant influence of water temperature on the occurrence of *Ae. albopictus* at oviposition sites, this effect vanished when different container types were analyzed separately. Therefore, this finding likely reflects temperature differences among container types rather than a contribution to inter-species differences. Overall, our findings are consistent with interspecific competition as the mechanism of species displacement. Whether the two species actively avoid laying eggs in the same breeding habitats due to species-specific semiochemical cues or whether one outcompetes the other in situ is yet to be addressed within Panama, but it has been observed that both species are rarely found together at the same oviposition site, even within areas of known species coexistence (Bennett et al. 2019a, b).

In addition, differences in the occurrence of *Ae. aegypti* and *Ae. albopictus* across a short-distance range (> 150 km), similar land use type, topography and altitude, yet sharp heterogeneous environment of the Azuero Peninsula (Fig. 2), suggests that their distributions are the result of shifting macroecological environmental factors shaping the outcome of interspecific competition. Niche modelling revealed that species displacement in the Azuero Peninsula has occurred at the niche edge where the environmental conditions are considered substandard for both *Ae. albopictus* and *Ae. aegypti*, suggesting that differences in species performance in the presence of a competitor influences species persistence under less than ideal conditions. In contrast, the niche modelling revealed that both species tend to occur throughout their core range in dry tropical regions of Panama. In support of this, the GLM for both species separately and for species co-occurrence supports that the presence of *Ae. aegypti* and *Ae. albopictus* are associated with higher temperatures and lower NDVI vegetation cover, i.e., a dry tropical environment, which encompasses a large portion of their range. Although both species occupy the same range of environmental values, the niche dynamics and the GLM analyses showed that the density of occurrence or association of *Ae. albopictus* was increased under wet tropical conditions (i.e., with higher rainfall and humidity) whereas *Ae. aegypti* was decreased. This finding is consistent with previous studies in other subtropical and tropical regions that show *Ae. aegypti* tends to inhabit dryer regions while *Ae. albopictus* is dominant under wetter climate conditions (Raharimalala et al. 2012; Hopperstad and Reiskind 2016). Therefore, interspecific competition acting in tandem with the environment provides a robust explanation for the observed differences in the geographical distribution and realized niche of *Ae. albopictus* and *Ae. aegypti* across Panama and more broadly.

It has been previously hypothesized that *Ae. albopictus* tends to occur alone in wetter and more vegetated environments while both *Ae. albopictus* and *Ae. aegypti* coexist in drier and warmer regions (Braks et al. 2003; Kaplan et al. 2010; Bagny Beilhe et al. 2012; Hopperstad and Reiskind 2016; Muzari et al. 2019). Our data fit this expectation, since the southwestern side of the Azuero Peninsula, where *Ae. albopictus* occurs alone, experiences a wet tropical climate. Conversely, *Ae. aegypti* persists in tandem with *Ae. albopictus* on the dry tropical eastern side of the Azuero Peninsula throughout the entire year. Here we note that both *Aedes* species co-occur in the eastern Azuero Peninsula, but never in the Southwest Azuero Peninsula within towns of similar levels of urbanicity and size. This further suggests that the environment may play an equal or larger role in determining species distribution differences than urbanicity at the macro-ecological scale. We do not expect the species distributions we have observed to be associated with recent trends of human-aided dispersal or insecticide treatment in the Azuero Peninsula, since all our localities are connected through a major highway, and no insecticide treatment has ever been systematically employed across this region. However, future studies will have to test the combined effect of the environment, urbanicity, vector control measures, and human-aided dispersal as drivers of local population dynamics in *Aedes* mosquitoes (Bennett et al. 2019c).

Both *Aedes* species have differences in their life history traits that could explain whether they are able to outcompete or persist with the other under different environmental conditions, i.e., under wet or dry tropical conditions. *Ae. aegypti* is able to tolerate dry environments better than *Ae. albopictus*. The eggs of *Ae. aegypti* are more resistant to desiccation and both the larvae and adults of *Ae. aegypti* have a higher thermal tolerance than *Ae. albopictus* (Juliano et al. 2002; Lounibos et al. 2010). Temperature and microhabitat characteristics such as local moisture supply and shade, are therefore likely to interact with these life history traits to determine species success. Temperature is known to influence the development time and survivorship of both *Ae. aegypti* and *Ae. albopictus*, although there is limited evidence that these parameters are altered under interspecific competition (Lounibos et al. 2002; Costanzo et al. 2005; Farjana et al. 2012). It has also been found that the larvae of *Ae. albopictus* outcompete *Ae. aegypti* within low nutrient environments but not when nutrients are abundant (Braks et al. 2003, 2004; Yee et al. 2004; Juliano 2010; Murrell and Juliano 2014). *Aedes aegypti* and *Ae. albopictus* develop in the same aquatic sites where they feed on microorganisms (e.g., bacteria) that can influence their life history. Recent findings from Panama indicate that *Ae. aegypti* and *Ae. albopictus* share a fairly similar niche in terms of the bacterial community they host at the larval and adult stages, except for the presence of some rare and unique bacterial community members in *Ae. aegypti* (Bennett et al. 2019a). That *Ae. aegypti* has a higher bacterial diversity than *Ae. albopictus* at the larvae and adult stages suggests it could be more a generalist aquatic feeder or has a higher tolerance of bacterial commensalism for which its members may have evolved specific functions (Minard et al. 2013). Nonetheless, that these mosquitoes tend to share a large proportion of bacterial types signals the need for further work to understand whether resource competition in association with bacterial acquisition, can impact on mosquito development and survival under different macroecological climatic conditions, i.e., temperature and rainfall (Kaplan et al. 2010; Bennett et al. 2019a). The surrounding vegetation, which contributes organic matter to larval nutrition within oviposition sites, is also another key environmental parameter impacting on interspecific competition (Reiskind et al. 2010, 2012). Certainly, it has been found that over large (Kraemer et al. 2015) and regional scales (Reiskind and Lounibos 2013) *Aedes* species distributions are determined by environmental variables including temperature, NDVI vegetation index, humidity and rainfall. In Florida *Ae. aegypti* is more abundant during the early rainy season than *Ae. albopictus*, which is more abundant during the late rainy and dry season (Reiskind and Lounibos 2013). This is not a pattern that extends to the tropical region of Panama, probably due to reduced diurnal and inter-seasonal variation compared to the subtropics.

### Implications of invasion by *Ae. albopictus* for the landscape of arboviral disease transmission

*Aedes aegypti* has been resident in Latin America since its invasive introduction from Africa during the 17th Century, and is considered the primary source of arbovirus transmission worldwide (World Health Organization 1997; Powell and Tabachnick 2013; Eskildsen et al. 2018). *Aedes albopictus* is a secondary vector, but its relevance for global arbovirus transmission has been amplified by the aggressive expansion of invasive populations from Asia during the 1980′s (Benedict et al. 2007). Globally, while *Ae. albopictus* has been implicated in several small outbreaks, the majority of dengue viruses (DENV 1,2,3,4) are thought to be transmitted by *Ae. aegypti*, due to its preference for both urbanized habitat (Alarcón et al. 2014; Leisnham et al. 2014) and human hosts (Ponlawat and Harrington 2005; Farjana and Tuno 2013). Likewise, while there have been a number of studies showing that although *Ae. albopictus* is biologically capable of transmitting DENV (Christofferson 2015), outbreaks that can be directly attributed to this species are rare (Gratz 2004; Effler et al. 2005; Paupy et al. 2011). *Aedes albopictus* is a biologically competent vector for a broader spectrum of viral pathogens than *Ae. aegypti*, including sylvatic zoonotic pathogens (e.g., Animal origin) cycling in forested habitats like YF, MAY and VEEV (O’meara et al. 1995; Bagny Beilhe et al. 2012; Leisnham et al. 2014; Pereira Dos Santos et al. 2018). This mosquito is considered an efficient bridge vector since it can pull pathogens from their sylvatic cycle in natural environments and boost their spill over opportunities into urbanized areas where humans are infected. In contrast, *Ae. aegypti* seems to play a larger role in the global transmission of urban re-emerging and emerging DENV, WNV, CHIKV and ZIKV (Leisnham et al. 2014).

*Aedes albopictus* have the potential to influence pathogen transmission both directly, by becoming a novel pathogen vector, or indirectly, by displacing or coexisting with *Ae. aegypti* (Bevins 2008). Thus far, the displacement of resident *Ae. aegypti* by the invasive and superior competitor *Ae. albopictus* has unknown consequences for arbovirus transmission risk across Panama. Given empirical outcomes from prior studies plus our current findings, we can postulate two indirect epidemiological scenarios on this regard. Firstly, in places of Panama where only *Ae. albopictus* occurs like in the southwestern part of the Azuero Peninsula, the potential for sylvatic disease spill over to humans can increase because the Asian tiger mosquito feeds on a wider range of vertebrate hosts and is competent to transmit a broader spectrum of emerging zoonotic pathogens than *Ae. aegypti* (Kilpatrick and Randolph 2012). Therefore, the establishment of *Ae. albopictus* as a bridge vector in rural areas of Panama, could facilitate the introduction of sylvatic MAY, VEEV and YF into the urban cities. At the same time though, the displacement of *Ae. aegypti* from rural areas could decrease the transmission risk of emerging and re-emerging DENV, WNV, ZIKV and CHIKV, as the latter focuses almost exclusively on humans for feeding and is a more efficient transmitter of these diseases. Secondly, in places of Panama where both *Aedes* species coexist like in the eastern part of the Azuero Peninsula, commensal *Ae. aegypti* could effectively maintain circulation of amenable zoonotic pathogens (e.g., YF, MAY and VEEV) transmitted to humans by *Ae. albopictus* while both species could sustain transmission of re-emerging and emerging DENV, WNV, ZIKV and CHIKV (Trpis and Hausermann 1978; Powell and Tabachnick 2013; Brown et al. 2014; McBride et al. 2014; Pereira Dos Santos et al. 2018).

Taken together, findings predict a more complicated and perhaps effective arbovirus transmission dynamics in areas of *Aedes* coexistence, including eastern Azuero Peninsula, Veraguas, Chiriqui, and Panama City. Nonetheless, how these two epidemiological scenarios may ultimately play out with regard to disease transmission dynamics still remains unclear in Panama as vector competence of MAY, VEE, YF, DENV, WNV, CHIKV and ZIKV is vector population and/or virus strain dependent (Díaz-Nieto et al. 2013; Gonçalves et al. 2014; Vega-Rúa et al. 2014; Liu et al. 2017). While, both mosquitoes have similar transmission efficiencies for CHIKV in Panama City (Vega-Rúa et al. 2014), their vector competencies have not been fully assessed in other areas of the country and for other arboviruses. Our findings call for various control strategies to be implemented to decrease arboviral transmission according to the presence of the different vector species in ecologically distinct areas of Panama. We also posit that vector control strategies specifically targeting *Ae. aegypti* (i.e., insecticide application inside houses or the release of Oxitec transgenic mosquitoes—OX513A), may not curb viral transmission in areas where both *Aedes* species coexist, or promote the proliferation of *Ae. albopictus* in areas of coexistence with unknown consequences for disease emergence. The long-term monitoring of *Ae. albopictus* and *Ae. aegypti* interactions will be crucial to continue assessing arbovirus transmission risk in Panama. Ascertaining species distribution data for *Aedes* vectors will also be essential to the modelling and prediction of disease spread and containment in the country. Future epidemiological studies in the Azuero Peninsula and across Panama will have to test whether or not our epidemiological predictions are correct.

## Supplementary Information

Below is the link to the Supplementary Information.

Supplementary file 1 (PDF 89 kb)

Supplementary file 2 (PDF 109 kb)

Supplementary file 3 (XLSX 232 kb)

Supplementary file 4 (XLSX 25 kb)

Supplementary file 5 (XLSX 10 kb)

## Data Availability

All data supporting the conclusions of this article are provided within the article and its additional files.
