# Supplementary material for: The role of heterogenous environmental conditions in shaping the spatiotemporal distribution of competing Aedes mosquitoes in Panama: implications for the landscape of arboviral disease transmission
Source: Biol Invasions. 2021 Mar 1;23(6):1933–48. doi: 10.1007/s10530-021-02482-y (PMC8550678; doi:10.1007/s10530-021-02482-y)
Supplement: Supplementary file 1 — (PDF 89 kb) [file 10530_2021_2482_MOESM1_ESM.pdf]

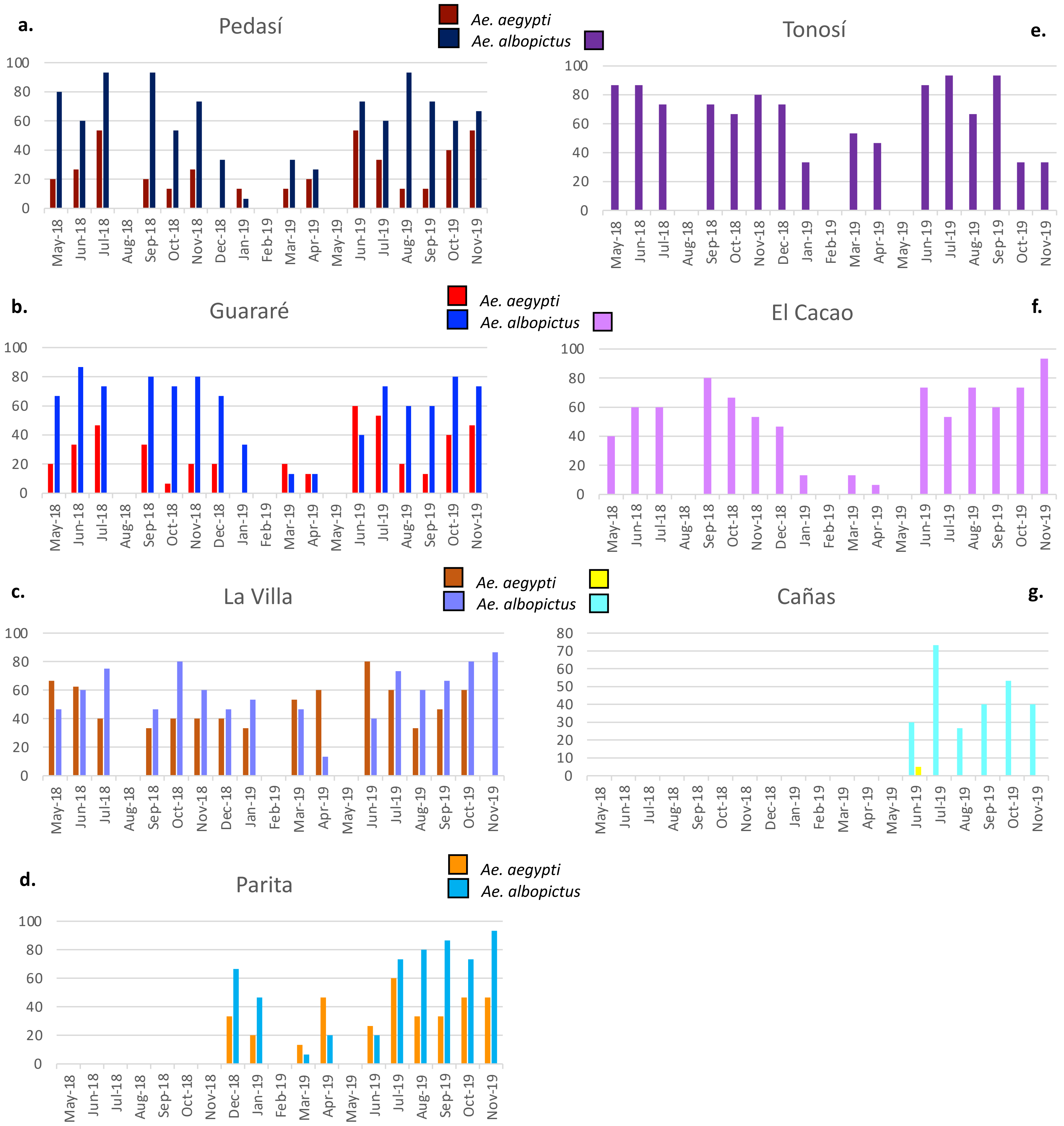

Supplementary Fig S1. The proportion of sampled sites positive for *Ae. aegypti* and *Ae. albopictus* at each location across each sampled month in 2018 and 2019 in a-d. the East Azuero Peninsula and e-g. Southwest Azuero Peninsula.
