# Supplementary material for: The role of heterogenous environmental conditions in shaping the spatiotemporal distribution of competing Aedes mosquitoes in Panama: implications for the landscape of arboviral disease transmission
Source: Biol Invasions. 2021 Mar 1;23(6):1933–48. doi: 10.1007/s10530-021-02482-y (PMC8550678; doi:10.1007/s10530-021-02482-y)
Supplement: Supplementary file 2 — (PDF 109 kb) [file 10530_2021_2482_MOESM2_ESM.pdf]

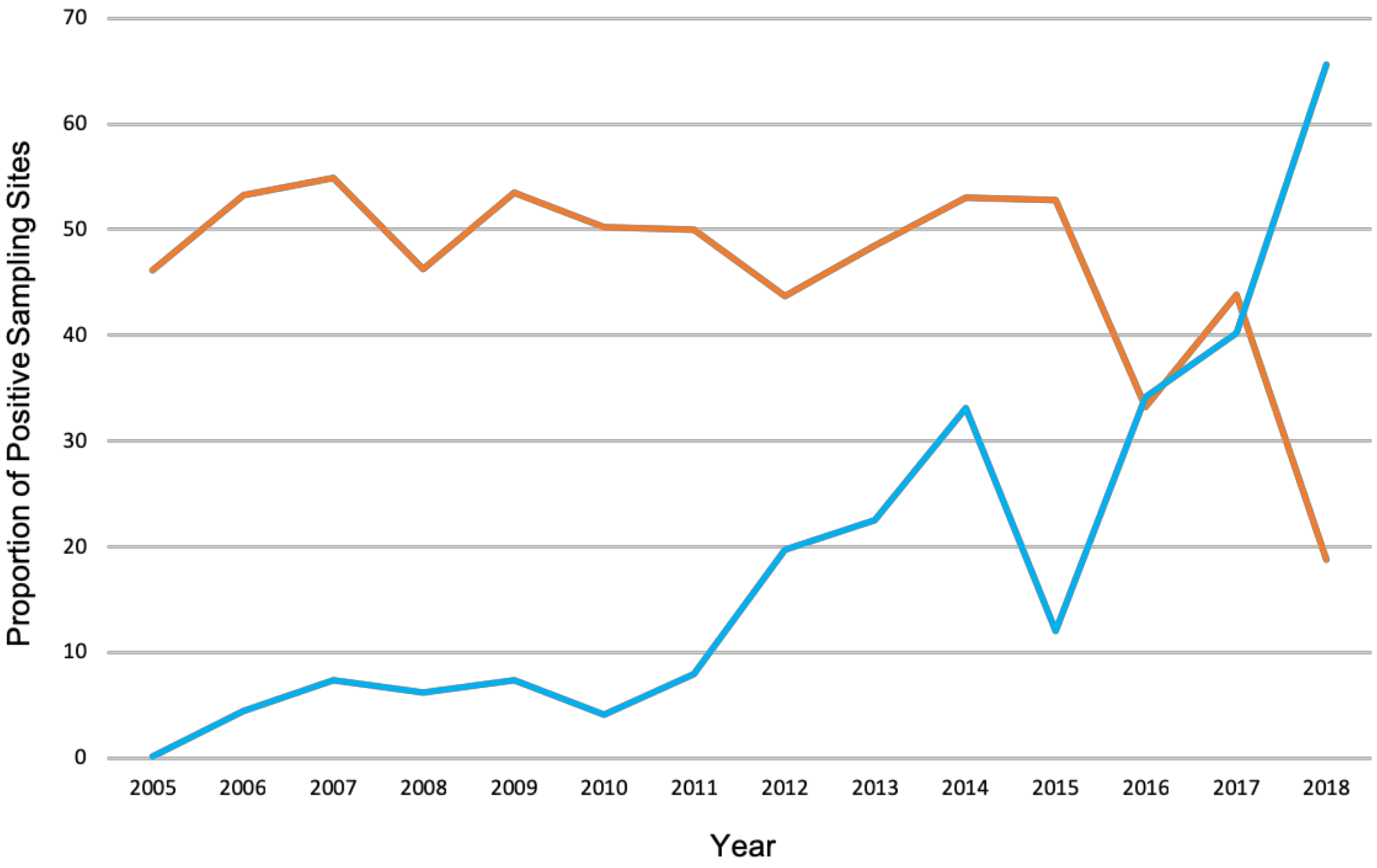

Supplementary Fig S2. The proportion of sampling sites positive for the presence of *Ae. aegypti* (orange) and *Ae. albopictus* (blue) across Panama from 2005 through 2018.
